# Free Zinc as a Predictive Marker for COVID-19 Mortality Risk

**DOI:** 10.3390/nu14071407

**Published:** 2022-03-28

**Authors:** Maria Maares, Julian Hackler, Alessia Haupt, Raban Arved Heller, Manuel Bachmann, Joachim Diegmann, Arash Moghaddam, Lutz Schomburg, Hajo Haase

**Affiliations:** 1 Department of Food Chemistry and Toxicology, Technische Universität Berlin, Straße des 17. Juni 135, 10623 Berlin, Germany; maares@tu-berlin.de (M.M.); alessia.haupt@campus.tu-berlin.de (A.H.); 2 TraceAge-DFG Research Unit on Interactions of Essential Trace Elements in Healthy and Diseased Elderly, Potsdam-Berlin-Jena, Germany; julian.hackler@charite.de; 3 Institute for Experimental Endocrinology, Charité-Universitätsmedizin Berlin, Corporate Member of Freie Universität Berlin, Humboldt-Universität zu Berlin, and Berlin Institute of Health, 10115 Berlin, Germany; raban.heller@med.uni-heidelberg.de; 4 Bundeswehr Hospital Berlin, Department of Traumatology and Orthopaedics, Septic and Reconstructive Surgery, 10115 Berlin, Germany; 5 Department of General Practice and Health Services Research, Heidelberg University Hospital, 69120 Heidelberg, Germany; 6 ATORG, Aschaffenburg Trauma and Orthopedic Research Group, Center for Orthopedics, Trauma Surgery and Sports Medicine, Hospital Aschaffenburg-Alzenau, 63739 Aschaffenburg, Germany; manuel.bachmann.md@gmail.com (M.B.); joachim.diegmann@klinikum-ab-alz.de (J.D.); 7 Orthopedic and Trauma Surgery, Frohsinnstraße 12, 63739 Aschaffenburg, Germany; email@arash.de

**Keywords:** trace element, free serum zinc, COVID-19, inflammation, survival

## Abstract

Free zinc is considered to be the exchangeable and biological active form of zinc in serum, and is discussed to be a suitable biomarker for alterations in body zinc homeostasis and related diseases. Given that coronavirus disease 2019 (COVID-19) is characterized by a marked decrease in total serum zinc, and clinical data indicate that zinc status impacts the susceptibility and severity of the infection, we hypothesized that free zinc in serum might be altered in response to SARS-CoV-2 infection and may reflect disease severity. To test this hypothesis, free zinc concentrations in serum samples of survivors and nonsurvivors of COVID-19 were analyzed by fluorometric microassay. Similar to the reported total serum zinc deficit measured by total reflection X-ray fluorescence, free serum zinc in COVID-19 patients was considerably lower than that in control subjects, and surviving patients displayed significantly higher levels of free zinc than those of nonsurvivors (mean ± SD; 0.4 ± 0.2 nM vs. 0.2 ± 0.1 nM; *p* = 0.0004). In contrast to recovering total zinc concentrations (r = 0.706, *p* < 0.001) or the declining copper–zinc ratio (r = −0.646; *p* < 0.001), free zinc concentrations remained unaltered with time in COVID-19 nonsurvivors. Free serum zinc concentrations were particularly low in male as compared to female patients (mean ± SD; 0.4 ± 0.2 nM vs. 0.2 ± 0.1 nM; *p* = 0.0003). This is of particular interest, as the male sex is described as a risk factor for severe COVID-19. Overall, results indicate that depressed free serum zinc levels are associated with increased risk of death in COVID-19, suggesting that free zinc may serve as a novel prognostic marker for the severity and course of COVID-19.

## 1. Introduction

The coronavirus disease 2019 (COVID-19) is caused by severe acute respiratory syndrome coronavirus 2 (SARS-CoV-2) [1]. While infection is diagnosed by the detection of the virus by polymerase chain reaction (PCR) or viral proteins by immunological methods in specimens obtained from the upper respiratory tract, further assessment of disease severity is performed using a number of laboratory markers [2]. Risk factors for severe infection with SARS-CoV-2 include age, male sex, certain comorbidities [3,4], and suboptimal micronutrient status [5,6]. Essential trace elements selenium, copper, and zinc, which are required for proper immune function, were widely discussed to impact susceptibility to COVID-19 and disease course [5,7,8,9].

Zinc is essential for innate and adaptive antiviral immunity [10]. While the underlying mechanisms of the antiviral response were found to be virus-specific, zinc was shown to be a critical factor in the progression of viral infections with hepatitis C or human immunodeficiency virus (HIV) [10], and was identified to be relevant for the direct antiviral defense against respiratory viruses, such as influenza and coronaviruses [10,11]. Regarding its role in SARS-CoV-2, zinc was found to not only improve the antiviral immune response, but also (i) prevent interaction of SARS-CoV-2 with the angiotensin-converting enzyme 2 (ACE2) on the cell membrane, (ii) affect cellular expression of ACE2, and (iii) impair viral replication (reviewed in [9,12]). Observational studies mostly comparing total serum zinc in mild, moderate, and/or severe COVID-19 patients provided strong indications that disease severity is associated with very low serum zinc levels [13,14,15,16,17,18,19,20,21].

Because zinc is a critical factor for various physiological processes, zinc deficiency has significant consequences for health. It is associated with a number of common diseases, critically impairs the immune system, and increases risk for infection and disease-related mortality [22,23]. To date, 16% of the world’s population is estimated to be zinc-deficient [24]. While severe zinc deficiency is characterized by low serum zinc levels, mild to moderate zinc deficiency is more difficult to detect and often stays unrecognized [22]. Causes of zinc deficiency are either insufficient zinc intake, or impaired intestinal absorption due to low bioavailability or zinc malabsorption diseases [4]. Accordingly, the risk of zinc deficiency is mainly determined by age, physiological status, or pathologic conditions [5,6]. Zinc supplementation can reverse symptoms of zinc malnutrition, and was discussed to decrease susceptibility to COVID-19 and improve convalescence [9,19]. 

Physiological total zinc concentrations in the sera of healthy humans range from 75 to 92 µg/dL, corresponding to 11.5–15 µM, representing less than 1% of total body zinc [25]. In serum, zinc is mainly bound to albumin and α-macroglobulin, which transport the trace mineral through the body [22]. Even though albumin in blood is not completely saturated, a subnanomolar fraction of zinc in serum exists that is considered to be free, also known as labile- or non-protein-bound zinc, respectively [26]. This zinc species interacts with low-molecular-weight ligands, such as amino acids or peptides, and is consequently defined as the available and biologically active zinc species in serum [27]. Free zinc in cells and body fluids is tightly regulated; fluctuations play an important role in intra- and extracellular signal transduction [28], and during viral recognition and antiviral response [10]. Accordingly, free zinc is discussed to be a suitable biomarker for alterations in zinc homeostasis and related diseases [29], and thus might be altered during SARS-CoV-2 infection. To test this hypothesis and investigate whether free serum zinc levels reflect disease severity, this study analyzed free zinc in the sera of a clinical cohort of survivors and nonsurvivors of COVID-19, for which changes of total serum zinc levels were available for comparison from our recent analysis [15]. 

## 2. Materials and Methods

### 2.1. Samples and Study Design

A cross-sectional study of patients with SARS-CoV-2 infection was conducted at nonprofit public Hospital Klinikum Aschaffenburg-Alzenau, Germany. A diagnosis of SARS-CoV-2 infection was confirmed by the positive detection of viral RNA using real-time PCR according to Corman et al. [30]. The study was conducted in accordance with the Declaration of Helsinki. Ethical counselling was provided by the authorities in Bavaria, Germany (Ethik-Kommission der Bayerischen Landesärztekammer, EA no. 20033), and the study was registered at the German Clinical Trial Register (Deutsches Register Klinischer Studien, ID: DRKS00022294). All enrolled patients or next of kin provided written informed consent. The mean number of samples taken per patient was 5.0 ± 4.3 (mean ± SD). Samples were stored at −80 °C (Aschaffenburg, Germany), and sent on dry ice to the analytical lab for trace element analysis (Institute for Experimental Endocrinology, Charité—Universitätsmedizin Berlin, Berlin, Germany). All measurements were conducted by scientists and technicians blinded to the clinical information. A set of commercially available human serum samples (*n* = 86, in.vent Diagnostica GmbH, Hennigsdorf, Germany) served as a reference cohort for healthy individuals.

### 2.2. Free Zinc Analysis

Free zinc levels in serum were determined using the low-molecular-weight fluorescent sensor Zinpyr-1 (Santa Cruz biotechnology, Dallas, TX, USA) as described [27]. To this end, 20 μL of serum, prediluted in assay buffer (1:10) and stored at −80 °C, was added to 80 μL prewarmed assay buffer containing Zinpyr-1 (final concentration 0.05 μM). Free zinc was determined on the basis of the fractional saturation of Zinpyr-1 using 15 µL EDTA or 800 µM ZnSO_4_ to induce a maximal and minimal fluorescence signal of the sensor, respectively [27]. The dissociation constant (K_d_) for the Zinpyr-1–zinc complex of 0.7 nM was then used for the calculation of free serum zinc concentrations [31,32].

### 2.3. Trace Element Analysis

Concentrations of total selenium, copper, and zinc in the serum samples were determined with total reflection X-ray fluorescence (TXRF) using a benchtop TXRF spectrometer (S4 T-STAR, Bruker Nano GmbH, Berlin, Germany) as previously described [15,33,34]. 

### 2.4. Serum Biomarker Quantification

Selenoprotein P (SELENOP) and ceruloplasmin (CP) had previously been determined in this set of samples as described [15,34]. In brief, SELENOP was quantified by using a validated commercial SELENOP-specific ELISA (selenOtest ELISA, selenOmed GmbH, Berlin, Germany). Quality of measurements was verified by including two human serum standards in each assay run, yielding an interassay CV of <15% during the analyses. Serum CP concentrations were determined by a recently developed sandwich ELISA including two monoclonal antibodies to human CP, as described [35]. A commercially available preparation of purified human CP (Ceruloplasmin 187-51-10, Lee Biosolutions, Maryland Heights, MO, USA) served as assay standard. 

### 2.5. Statistical Analysis

Statistical analysis was conducted using GraphPad Prism software version 9.3.1 (GraphPad Software Inc., San Diego, CA, USA). Data were tested for normal distribution with the Shapiro–Wilk test. Correlations were analyzed with Spearman correlation analysis. Differences between two groups were tested with the Mann–Whitney U test, and more than two groups were compared with the Kruskal–Wallis test followed by Dunn’s multiple-comparison tests. All tests were two-tailed. Differences were assumed to be significant with *p* values * *p* < 0.05, ** *p* < 0.01, or *** *p* < 0.001, as indicated in the figure legends.

## 3. Results

### 3.1. Patient Characteristics 

In this observational study, serum samples (*n* = 169) of COVID-19 patients were studied with regard to their serum levels of essential trace elements, including total serum zinc and free zinc concentrations, along with two biomarkers of selenium (total serum Se and SELENOP) and two biomarkers of copper status (total serum copper and CP concentrations). The analyzed serum samples were consecutively collected from 33 COVID-19 patients during their hospitalization, over a time course of up to 48 days. However, as the serum samples of COVID-19 patients were limited, only 167 of the initial 169 had sufficient remaining volume available to allow for the analysis of free zinc in this study. Patients were divided into two groups: patients that survived the disease and were discharged (*n* = 27) or nonsurvivors (*n* = 6), as presented in Table 1. A set of commercially available sera (*n* = 86) from subjects with a self-assessed status as healthy served as reference (CTR). 

### 3.2. Free Zinc and Total Trace Element Status in Serum of COVID-19 Patients 

**Figure 1:**
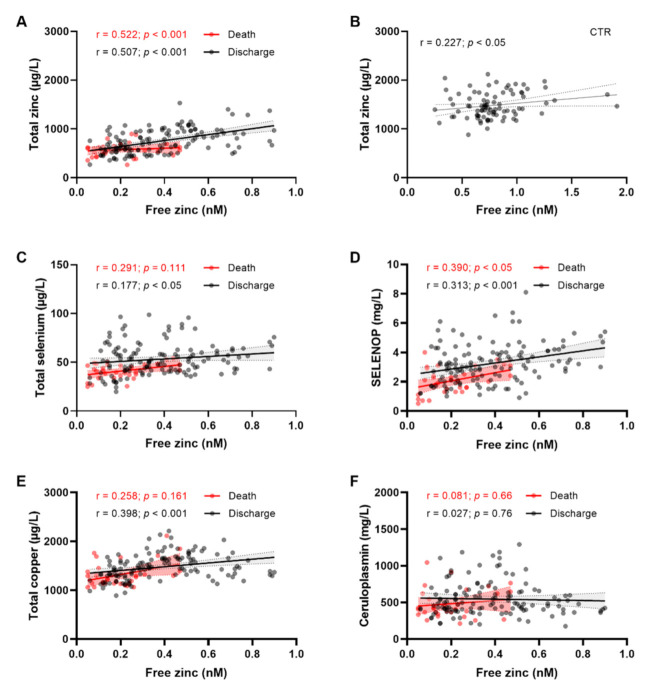
Free zinc in relation to total zinc and biomarkers of selenium and copper status in sera from COVID-19 patients. COVID-19 serum samples are separated and color-coded into patients that had died from COVID-19 (red, denoted as “Death”), and those who had survived (black symbols, “Discharge”). Free serum zinc concentrations were compared to (**A**) total zinc in COVID-19 patients, (**B**) total zinc in control subjects (CTR), (**C**) total Se in patients, (**D**) serum SELENOP in patients, (**E**) total serum copper concentrations, and (**F**) serum ceruloplasmin concentrations. Spearman correlation coefficients (r) and *p* values are indicated.

To investigate if the level of bioavailable free zinc species in serum was affected during COVID-19, free zinc concentrations in serum of COVID-19 survivors and nonsurvivors were compared. The concentrations of free zinc correlated strongly and significantly with total serum zinc concentrations in COVID-19 patients (Figure 1A, Spearman correlation coefficient; death r = 0.522, discharged r = 0.507, *p* < 0.001). Less stringent correlation was observed between total and free zinc concentrations in healthy subjects (r = 0.227, *p* < 0.05) (Figure 1B). The selenium status biomarkers total selenium (Figure 1C) and serum SELENOP (Figure 1D) showed similar positive associations with free zinc concentrations, with SELENOP displaying the more stringent correlations in both survivors (Discharge) and nonsurvivors (Death). Total copper concentrations were positively associated with free zinc in survivors only, but not in the group of samples from nonsurvivors (Figure 1E). Ceruloplasmin appeared unrelated to free zinc concentrations, both in survivors and nonsurvivors (Figure 1F). 

### 3.3. Free Serum Zinc Concentrations in Relation to Survival 

**Figure 2:**
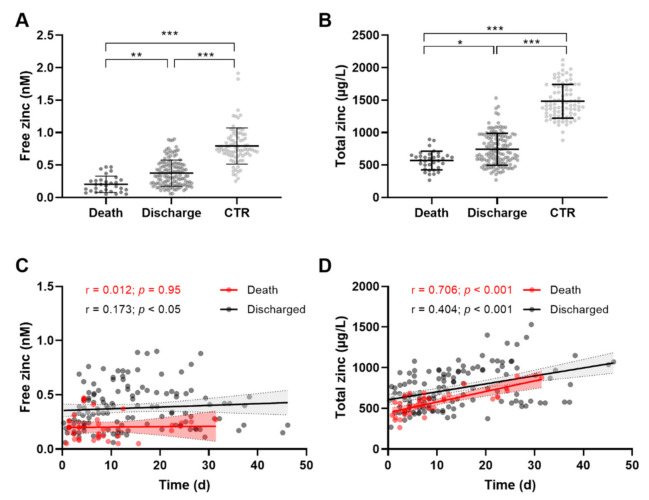
Dynamics of free serum zinc and total serum zinc during COVID-19. (**A**) Free zinc and (**B**) total zinc concentrations in sera of COVID-19 patients, separated with regard to patients that survived (“Discharge”) or died (“Death”), in comparison to serum of healthy controls (CTR). Scatter plots depict changes in (**C**) free and (**D**) total serum zinc concentrations over time during hospital stay. Data are presented as means ± SD, and significant differences are indicated (Kruskal–Wallis test followed by Dunn’s multiple–comparison test (* *p* < 0.05; ** *p* < 0.01; *** *p* < 0.001) (**A**,**B**)), and as scatter plots (**C**,**D**), including Spearman correlation coefficients (r) and *p* values. Data presented in (**B**) are shown for comparison and were published previously [15].

Sera of healthy controls contained 0.8 ± 0.3 nM free zinc (Figure 2A). Depending on the severity of COVID-19, free serum zinc showed significantly decreased concentrations, with 0.2 ± 0.1 nM free zinc in nonsurvivors versus 0.4 ± 0.2 nM in those that survived the infection and were discharged from the hospital (Figure 2A). Total serum zinc was significantly lower in COVID-19 patients than that in the CTR group, and levels in sera of nonsurvivors were lowest (Figure 2B, mean ± SD, death 568.8 ± 144.3 µg/L, discharge 742.9 ± 246.4 µg/L, CTR 1482 ± 261.1 µg/L), as recently described in the same cohort [15]. Similar to the free zinc concentrations, total serum zinc correlated inversely with disease severity (Figure 2B). Notably, the decreased status in free serum zinc in COVID-19 patients compared to that of CTR subjects was more pronounced than that of total serum zinc. Free and total serum zinc in survivors were decreased by approximately 50%, but free serum zinc in nonsurvivors was only 25% of that in healthy CTR subjects, and total serum zinc was decreased to 38% in comparison to CTR sera (Figure 2A,B). No difference in free zinc levels was observed with disease duration in either the sera of the discharged patients or in the group of nonsurvivors (Figure 2C). Total serum zinc levels of COVID-19 patients increased significantly with disease duration (Figure 2D, death: r = 0.706, *p* < 0.001; discharged: r = 0.404; *p* < 0.001), particularly in sera of patients that did not survive, which is in contrast to the dynamics of free zinc levels (Figure 2C). 

### 3.4. Sex-Specific Differences in Free and Total Serum Zinc Concentrations during COVID-19

**Figure 3:**
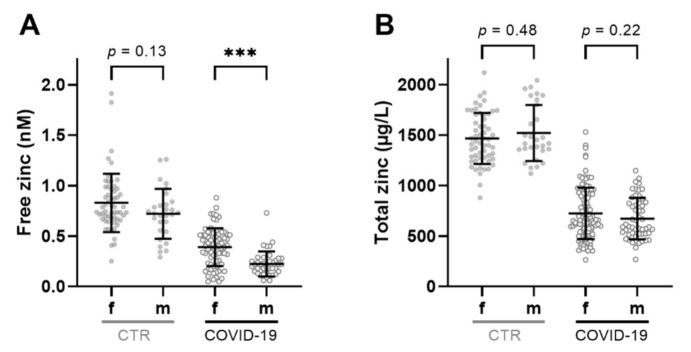
Impact of sex on free and total serum zinc concentrations. (**A**) Free zinc and (**B**) total zinc concentrations in sera of male (m) and female (f) COVID-patients and CTR subjects. Data are shown as means ± SD, and comparisons were analyzed by Mann–Whitney U test (*** *p* < 0.001).

There was no sex-dependent difference in free serum zinc concentrations in the CTR group (Figure 3A). COVID-19 appeared to affect the amounts of free serum zinc differently in female and male patients, with the latter displaying significantly lower free zinc concentrations than those of females (Figure 3A). These differences were not observed for total serum zinc in either the sera of healthy subjects or in COVID-19 sera (Figure 3B).

### 3.5. Dynamics of Total Copper/Zinc and Total Copper/Free Zinc Ratio in Serum of COVID-19 Patients

**Figure 4:**
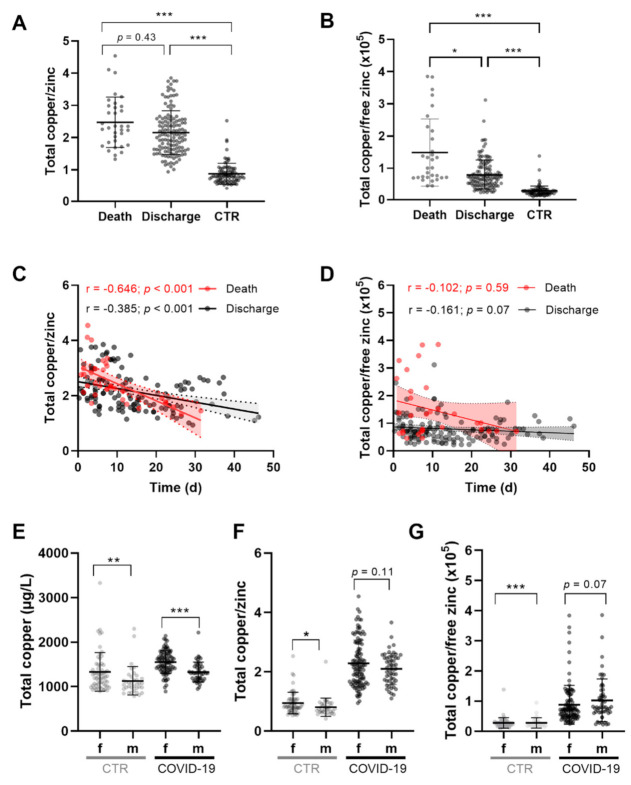
Analysis of total copper, copper/zinc, and copper/free zinc in serum of patients and controls. Analysis of (**A**) ratio of total copper and zinc concentrations, (**B**) total copper and free zinc, (**C**,**D**) dynamic changes in COVID-19, and (**E**–**G**) comparison of patients with healthy controls (CTR). (**A**) Total serum copper/zinc concentrations were elevated in COVID-19 patients as compared to controls. (**B**) Nonsurvivors displayed particularly elevated total copper/free zinc ratio in comparison to those of survivors and controls. (**C**) Total copper/zinc decreased in serum of COVID-19 patients during hospitalization. (**D**) Total serum copper/free zinc displayed only slight alterations with time. Consistent sex-specific differences were observed for (**E**) total serum copper, (**F**) total copper/zinc, and (**G**) total copper/free zinc concentrations in control subjects. Significant difference between male and female COVID-19 patients was observed for (**E**) total copper and (**F**) total copper/zinc, but not for (**G**) total copper/free zinc. Differences between two groups were tested with Mann–Whitney U test, and more than two groups were compared with Kruskal–Wallis test, followed by Dunn’s multiple comparison tests (* *p* < 0.05; ** *p* < 0.01; *** *p* < 0.001). Spearman correlation coefficients (r) are indicated.

Total serum copper to total zinc (Cu/Zn) ratio in the serum of COVID-19 patients was significantly and more than twice higher than that in the serum of control subjects (Figure 4A; death 2.5 ± 0.8, discharge 2.2 ± 0.7, CTR 0.9 ± 0.3). Comparing Cu/Zn concentrations in the sera of patients, this ratio was slightly higher in nonsurvivors than that in survivors. The difference was even more pronounced for the ratio of total copper and free zinc (Cu/free Zn), which was 1.9 times higher in serum of nonsurvivors than that in survivors (Figure 4B, death 1.49 ± 1.04, discharge 0.8 ± 0.46, CTR 0.23 ± 0.17). Correlation analysis showed a negative relationship between Cu/Zn and disease progression. This negative relationship was stronger in patients who did not survive COVID-19 (Figure 4C, death r = −0.646 vs. discharge r = −0.385). Parameters Cu/free Zn and disease progression showed a less stringent correlation (Figure 4D, death r = −0.102, discharge r = −0.161). This ratio appeared to slightly decrease with disease duration in nonsurvivors, while it did not change in the serum of discharged patients (Figure 4D). Serum copper concentrations of women were significantly higher than those of male patients (Figure 4E; *p* < 0.001), yet there was no sex-dependent difference in serum Cu/Zn or Cu/free Zn in the COVID-19 patients (Figure 4F,G). Female samples of the CTR group showed significantly higher Cu/Zn and Cu/free Zn ratio, and total copper levels than those of the serum samples from men (Figure 4E–G).

## 4. Discussion

This study describes alterations in trace elements in healthy subjects and patients with COVID-19 under specific consideration of the zinc status measured as either total serum concentrations or as free zinc levels. Results are related to other trace elements, namely, selenium and copper, where two biomarkers each were available for direct comparison. Data indicate significant but weak correlation of free zinc concentrations with total serum zinc in healthy controls, and more stringent correlation in patients with COVID-19. Notably, minute levels of free zinc only were detectable in the most severely affected patients who did not survive the disease, with male patients displaying stronger deficits compared to those of female patients. In comparison to healthy controls, free zinc appears to decline with disease severity, thereby providing some predictive information on disease course and mortality risk in COVID-19.

In previous studies, COVID-19 was characterized by a marked decrease in total serum zinc, and clinical data so far indicate that the individual zinc status affects susceptibility, severity, and mortality related to the infection [13,14,15,16,17,20,21]. The current study adds novel and relevant information on zinc status, and demonstrates that COVID-19 is also associated with a severe decrease in the readily available free zinc pool in serum. On the basis of in vitro studies, free zinc was suggested to provide potent antiviral activity [36]. The data obtained in the present study agree with this notion, in particular with respect to nonsurvivors, as male patients were displaying the lowest free zinc levels. However, due to its observational design, no mechanistic conclusions can be drawn from our data. The small number of patients, and the unequal distribution of the number of survivors and nonsurvivors must also be considered to be limitations of the present study. 

Free zinc represents only a minimal fraction of total serum zinc, with free zinc concentrations being in the lower nanomolar range [27]. In healthy subjects, the correlation between free and total serum zinc was only moderate. This suggests that free zinc is not merely a subset of total serum zinc. It rather appears to be an independent pool constituting the fraction of total zinc with higher availability and biological activity [27]. During COVID-19 infection, however, the free serum zinc pool decreased strongly and correlated tightly with the declined total serum zinc both in survivors and nonsurvivors. This notion highlights that, in pathophysiology, where total zinc may decrease below the lower limit of the reference range of 642.5 μg/L, both fractions of serum zinc become depleted, independent of binding state [15]. Although a reference range for free zinc remains to be established by larger cohort studies, the relative decrease in zinc concentrations in nonsurvivors compared to healthy subjects was even more pronounced for free zinc compared to total zinc. Albumin is the main zinc binding and buffering protein in serum, and hypoalbuminemia is associated with severe COVID-19 [37]. Hence, the total zinc binding capacity of serum decreases with declining albumin concentrations, and should lead to an increase in free zinc. However, this was not observed in the sera of COVID-19 patients. Instead, there was a parallel decline in both serum zinc biomarkers, consistent with observations in a porcine sepsis model, where a decrease in both free and total serum zinc concentrations along with declining serum zinc-binding capacity was reported [38]. 

Total serum zinc is still most commonly used to determine zinc status, even though it is not the ideal biomarker to identify an individual’s zinc status, particularly for mild zinc deficiency [27]. Although the suitability of free zinc as a biomarker requires further investigation, the results of this study indicate that it might be useful to detect pathological changes in zinc homeostasis. Recently, the zinc status index (ZSI), consisting of the linoleic acid:dihomo-γ-linolenic acid ratio, mRNA expression of zinc-related proteins, and gut microbiome profile, was suggested as a suitable alternative to detect small alterations of zinc status [39]. In connection with COVID-19, metabolomics were also suggested to serve as a sensitive approach to detect micronutrient deficiencies and monitor trace element imbalances during disease progression [40]. Individual metabolic profiles were used to monitor malnutrition of macro- and micronutrients, such as calcium [41]. This method could probably give a general overview on systemic changes resulting from zinc deficiency, this being a functional approach for detecting the consequences of micronutrient insufficiency. While it has not yet been established for human zinc deficiency, it was discussed to serve as a valuable platform to detect severe zinc deficiency in rats [42]. 

With results derived from observational studies such as ours, it is not possible to assess whether (a) a pre-existing zinc deficiency promoted infection with SARS-CoV-2 or (b) low serum zinc levels are a direct consequence of COVID-19 and the associated hypoxia, immune response, and inflammation. In addition, the influence of trace elements should never be considered in isolation, as these essential micronutrients always act in synergy in the different phases of the immune response [43]. A zinc deficit impairs both the adaptive and the innate part of the immune system, thereby increasing the risk of infection. Given that an imbalanced immune system was coined as one of the hallmarks of COVID-19 [9], and zinc was identified to affect susceptibility to infection with SARS-CoV-2 [9], a low zinc status may directly contribute to the risk of COVID-19 and a severe disease course. However, serum zinc might also decline due to systemic inflammation, which is documented in sepsis, surgical stress, and critically ill patients [38,44,45]. This process, known as nutritional immunity, aims to starve pathogens by shifting zinc to the liver, and is characterized by increased interleukin (IL)-6 secretion by macrophages, which upregulates hepatocellular metallothionein and Zrt-, Irt-like protein (ZIP)14, supporting zinc transport into hepatocytes [46,47]. While nutritional immunity is well-described during microbial infections and sepsis [38,48], it is not well-characterized for viral diseases. However, serum IL-6 levels are dramatically elevated and are associated with disease severity and mortality risk in COVID-19 [49,50]. 

Sex-dependent characteristics in trace-element homeostasis affecting serum levels are mainly known for selenium and copper [51,52]. So far, sex-specific differences in free serum zinc levels were observed in humans and mice [27,53]. This needs to be considered when identifying predictors for diseases, remarkably because male sex was suggested as a risk factor for COVID-19 morbidity and mortality [54]. Accordingly, free serum zinc levels of male COVID-19 patients were lower than those in females, whereas total zinc levels and free serum zinc in healthy subjects did not differ between sexes. Likewise, the Cu/Zn ratio did not indicate sex-specific differences, but total copper levels in the serum of female patients were more strongly elevated than those in male patients. The latter was also observed in healthy adult women, who have significantly higher serum copper concentrations than those of men, which was also reflected by a higher serum Cu/Zn ratio [51]. 

Total serum zinc levels and the Cu/Zn ratio are common markers to identify the severity of inflammation, particularly in the acute phase of infectious diseases and in critically ill patients [38,45,55,56]. Cu/Zn ratio is rooted in diminished serum zinc and/or increase in serum copper levels [57], and was proposed to be a better prognostic marker for pathological conditions than total serum copper or total serum zinc levels alone are [58]. In the serum of COVID-19 patients, elevated Cu/Zn ratio was observed, in line with prior reports [14,18], and it was suggested to relate to COVID-19 severity and survival [14]. Likewise, the ratio of total copper to free zinc in the serum of COVID-19 patients was profoundly increased and reflected the odds of survival. Regarding the suitability of serum zinc levels as a marker for COVID-19, the two measured parameters of zinc status in the serum of COVID-19 patients both distinctly decreased with severity of the disease. Total zinc had been suggested to serve as prognostic indicator for COVID-19 severity [15,17], particularly in combination with SELENOP status [15]. However, with it being the biologically active zinc species in blood, free zinc might be considered to be an additional and potentially very meaningful and sensitive parameter for zinc homeostatic alterations in critically ill patients, which might also explain the more pronounced decline in free zinc in the serum of nonsurvivors compared to total zinc levels. In contrast to total zinc, free zinc levels in serum of patients did not improve during hospital stay, particularly for nonsurvivors. Thus, free zinc might be a suitable prognostic marker for the severity of COVID-19, particularly for outcome prediction [2]. 

This conclusion, however, remains to be confirmed in more extensive and larger studies that include data on patient characteristics such as comorbidities associated with alteration of zinc homeostasis and immune response. Since it was discussed that zinc supplementation might reduce the incidence of infection and support the convalescence in COVID-19 [9,19], further analyses should also aim to investigate whether the declining free zinc pool in the serum of COVID-19 patients can be rescued by zinc administration. A first promising intervention study in COVID-19 patients with severe acute distress syndrome supports the notion on positive effects of supplemental selenium and zinc for convalescence [7]. From a technical point of view, the assessment of free zinc concentrations offers certain advantages over total serum zinc analyses, as the experimental procedure is straightforward, can be established quickly in hospitals, and requires only a small amount of serum sample [27]. The fluorometric assay does not require costly and maintenance-intensive instruments, such as inductively coupled plasma mass spectrometry (ICP-MS) or flame atomic absorption spectrometry (FAAS), but solely relies on a fluorescence plate reader. Hence, it could be developed into a point-of-care test and support fast bedside analytics, thereby supporting the essential diagnostics in the intensive care unit (ICU). An additional, novel, and likely meaningful parameter of trace element status and micronutrient deficiency would become detectable and accessible for therapeutic supplemental correction in order to support the immune system in particularly challenging conditions such as severe disease.

## 5. Conclusions

In this study, free serum zinc was identified as a promising prognostic parameter for COVID-19 severity. Further clinical studies are required to highlight the importance of free serum zinc as a meaningful and readily accessible marker for assessing COVID-19 severity and progression. 

## Figures and Tables

**Table 1:** Overview of patients and serum samples in this study.

	COVID-19 Patients			CTR Sera
	Death *	Discharge	Total	
Female	4 (67%)	15 (56%)	19 (58%)	57 (66.3%)
Male	2 (33%)	12 (44%)	14 (42%)	29 (33.7%)
**Age (median, IQR)**				
Female	89 (89; 89)	79 (64; 82)	82 (75; 89)	35.0 (24; 42)
Male	92 (81; 92)	78 (61; 82)	81 (65; 82)	34.0 (26; 47)

* Death associated with COVID-19 diagnosis; IQR, interquartile range.

## Data Availability

Anonymized data are available from the corresponding author upon reasonable request.
